# Deep learning-based automated guide for defining a standard imaging plane for developmental dysplasia of the hip screening using ultrasonography: a retrospective imaging analysis

**DOI:** 10.1186/s12911-025-02926-8

**Published:** 2025-02-18

**Authors:** Kyung-Sik Ahn, Ji Hye Choi, Heejou Kwon, Seoyeon Lee, Yongwon Cho, Woo Young Jang

**Affiliations:** 1 Department of Radiology, Korea University Anam Hospital, 73, Goryeodae-ro, Seoungbuk-gu, Seoul, 02841 Republic of Korea; 2 Advanced Medical Imaging Institute, Korea University Anam Hospital, 73, Goryeodae-ro, Seoungbuk-gu, Seoul, 02841 Republic of Korea; 3 Department of Computer Science and Engineering, Soonchunhyang University, Asan-si, South Korea Republic of Korea; 4 LG Electronics, 19, Yangjae-daero 11-gil, Seocho-gu, Seoul, Republic of Korea; 5 Department of Biomedical Engineering, Korea University, Goryeodae-ro, Seoungbuk-gu, Seoul, 02841 Republic of Korea; 6 Department of Orthopedic Surgery, Korea University Anam Hospital, 73, Goryeodae-ro, Seoungbuk-gu, Seoul, 02841 Republic of Korea

**Keywords:** Convolutional neural network, Deep learning, Developmental dysplasia of the hip, Ultrasonography

## Abstract

**Background:**

We aimed to propose a deep-learning neural network model for automatically detecting five landmarks during a two-dimensional (2D) ultrasonography (US) scan to develop a standard plane for developmental dysplasia of the hip (DDH) screening.

**Method:**

A model of global and local networks was developed to detect five landmarks for DDH screening during 2D US. Patients (*N* = 532) who underwent hip US for DDH screening from January 2016 to December 2021 at a tertiary medical center were enrolled. All datasets were randomly split into training, validation, and test sets in a 70:10:20 ratio for the final assessment of landmark detection. The performance of this model for detecting five landmarks for guiding DDH was analyzed using the root mean square error (RMSE) and dice similarity coefficient.

**Results:**

The RMSE value for the five landmarks for diagnosing and classifying DDH using global and local networks was 4.023 ± 3.723. The point results using EfficientNetB2 were 1.69 ± 1.26 (first point), 3.34 ± 2.37 (second point), 2.54 ± 1.61 (third point), 5.92 ± 4.25 (fourth point), and 6.61 ± 4.82 (fifth point).

**Conclusions:**

Our deep-learning network model is feasible for detecting five landmarks for DDH using ultrasound images. The primary parameters to determine DDH will be significantly detected by applying the deep-learning model in clinical settings.

## Background

Developmental dysplasia of the hip (DDH) is a spectrum of neonatal hip developmental abnormalities including congenital dislocation, subluxation, and dysplasia of the hip. Early DDH detection is essential as it allows for the implementation of noninvasive treatment methods such as the Pavlik harness in order to achieve good long-term results [1, 2]. However, neglected DDH cases need extensive surgical treatment and show poorer prognosis, with a lifelong impact on gait and possible need for joint replacement. Considering the high cost of neglected DDH cases compared to those with early treatment, the timely detection of DDH is a concern for pediatric orthopedics and has a potential societal impact. Multiple countries are considering a newborn hip screening program suited to each country’s medical infrastructure resources for detecting DDH. Some countries sponsor universal screening of any newborn around the age of six weeks, due to a meta-analysis reporting that the incidence of late-diagnosed DDH can be significantly decreased when universal hip screening is adopted compared with selective hip screening [3]. In children below the age of six months, the femoral head is not yet ossified and does not appear in plain radiographs. Thus, ultrasonography (US) is accepted as a reliable tool for the early detection of DDH since it is noninvasive and can identify problems in the cartilaginous hip much earlier than radiography [4].

However, due to the nature of US, which is highly dependent on the operator, hip US should be performed by well-trained doctors [5] who accurately understand the Graf [6] or Harcke methods [7]. Harcke et al. suggested that hip US should be performed by a physician who has examined at least 100 infants aged < 6 months. Measurement in an inappropriate imaging section is meaningless and irrelevant for diagnosis or treatment. To obtain a reproducible ultrasonographic plane through the hip joint, appropriate scan sectioning should be addressed. The implementation of Graf’s method poses significant challenges and relies heavily on operator proficiency due to various factors, such as suboptimal image resolution, anatomically insufficient visuals, substantial variation in the configuration of pertinent anatomical landmarks, and inadequate training and expertise. Multiple studies have demonstrated notable inter-operator variability, potentially leading to discrepancies in diagnosis between dysplastic and normal cases, or altering the severity of dysplasia, consequently influencing treatment recommendations. Notably, the intra- and interobserver error is lower for normal hip evaluations but tends to be higher for borderline and abnormal hip assessments [8, 9]. This is one of the reasons that make US examination difficult, especially by an inexperienced examiner.

When performing hip US, if an adviser guides the location of the probe on the midline of the acetabulum, obtaining the standard imaging plane by moving the probe to the correct direction would be helpful. In particular, the following three landmarks in coronal view are used to define the standard plane for infant hip US: straight iliac line, tip of the acetabular labrum, and transition from the os ilium to the triradiate cartilage.

A deep-learning neural network model system can support orthopedic surgeons in medical fields, thus decreasing interobserver variability and misdiagnosis of DDH. Several deep network neural model systems for evaluating DDH have been reported. However, all previous systems focused on measuring alpha and beta angles and did not assess the appropriateness of the plane location, i.e., whether the ultrasound image is in the anterior, standard, or posterior plane [10, 11]. A previous study introduced a check angle to determine whether the plane was appropriate, but it did not distinguish between the anterior and posterior planes; moreover, that study also focused more on the evaluation of DDH after ultrasound image acquisition [12]. Furthermore, previous systems do not provide real-time US guidance. Since obtaining a reproducible standard plane through the hip joint is crucial for decreasing interobserver error, this study aimed to evaluate the performance of a deep-learning neural network model with real-time detection of five landmarks during two-dimensional (2D) US scan to achieve a standard plane for DDH evaluation.

## Materials and methods

The study protocol was approved by the institutional review board for human investigations at the Korea University Anam Hospital (KUAH), and the requirement for informed consent was waived owing to the retrospective design of our study. Datasets were deidentified for preserving patient privacy. All processes were performed according to relevant regulations and guidelines.

### Datasets

From January 2016 to December 2021, US images of patients screened for DDH were analyzed. The inclusion criteria were: coronal plane images of the hip and US images of patients aged < 8 months. Meanwhile, transverse plane and suboptimal images with blurring were excluded. The inclusion and exclusion of the images were confirmed by an experienced radiologist. Altogether, 2,823 consecutive US images were obtained from 532 infants (male: female = 223:309, average age 5.95 ± 8.72 months, 10.16 ± 8.08 days) from 2016 to 2021. All images were obtained using one of the following devices: Philips iU22 (Philips Healthcare, Eindhoven, The Netherlands), Aplio 500 (Canon Medical Systems, Otawara, Japan), or Aplio i700 (Canon Medical Systems). The images were anonymized and documented as Digital Imaging and Communications in Medicine files. Patient information at the top of the US images was collectively cropped. All datasets were randomly split into training, validation, and test sets in a 7:1:2 ratio for the final assessment of landmark detection (Table 1).

**Table 1:** Number of ultrasonography images for the final assessment of landmark detection

Type	Number of images	
	Training set (Validation set)	Test set
Ultrasonography	2033 (225) images	565 images
Male	200 patients	23 patients
Female	278 patients	31 patients
Total	2033 (225) images 478 patients	565images 54 patients

Note: Images indicate the number of ultrasonography sessions performed by the probe of the ultrasonic machine

### Labeling

**Fig. 1:**
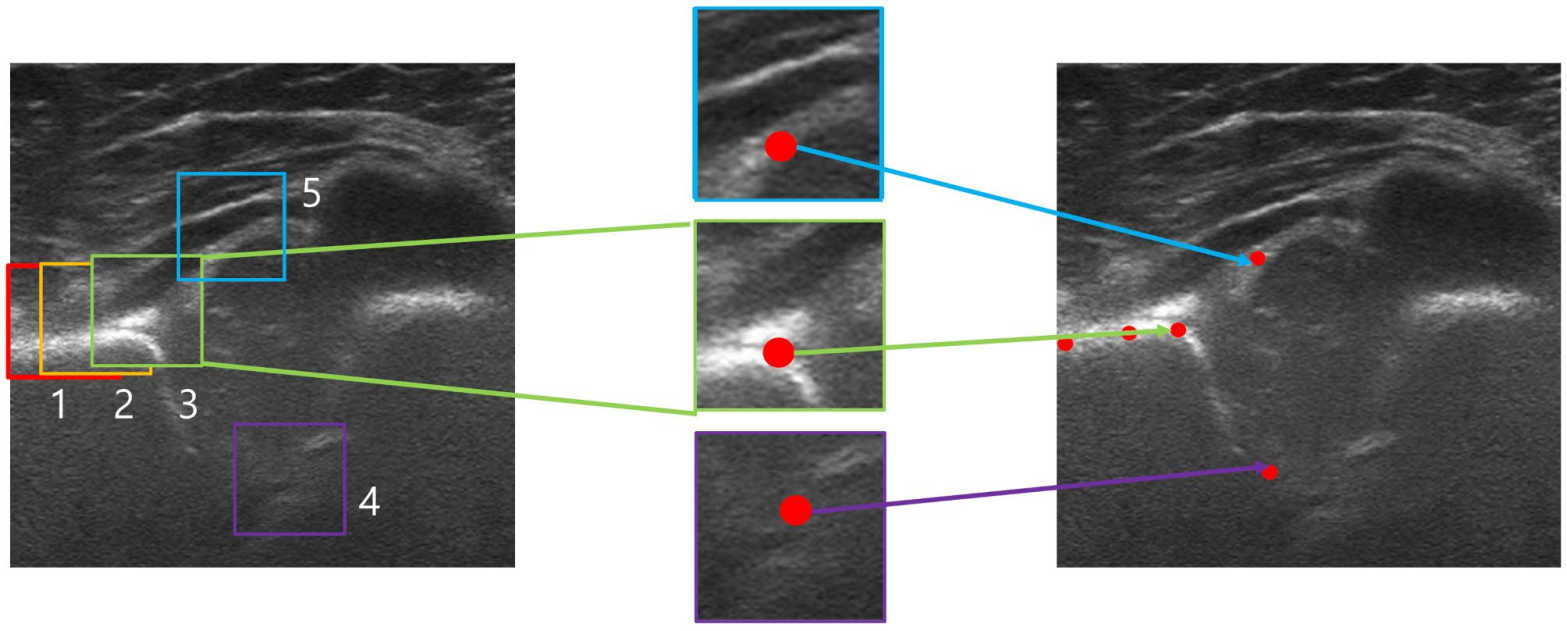
The five landmarks were defined as follows: the lower edge of the iliac bone, midpoint of the iliac bone, acetabular bone edge, triradiate cartilage, and the end of the acetabular labrum

Five landmarks were defined as follows: (1) lower edge of the iliac bone, (2) midpoint of the iliac bone, (3) acetabular bone edge, (4) triradiate cartilage, and (5) end of the acetabular labrum (Fig. 1).

The first three landmark represent a straight iliac line and determine the appropriateness of the plane. When employing the standard vertical projection in posterior hip plane, the depiction of the iliac silhouette reveals a concave contour. This concavity corresponds to the gluteal fossa located in the posterior region of the iliac wing. Additionally, the bony rim exhibits a rounded or “nose-shaped” appearance. Therefore, in the posterior plane, the third landmark of the acetabular bone edge should protrude as the bony rim exhibits a nose-shaped appearance and interrupts the straight iliac line. Additionally, in the anterior sectional plane, the iliac bone bends towards the probe. In our study, if the absolute value of the angle between the lines connecting points 1 and 2 or 2 and 3 was less than 5 degrees, the iliac line was considered to be straight and the imaging plane assumed to be appropriate. The fourth and fifth landmarks were checked for measuring alpha and beta angles consecutively, according to the iliac wing representation by the first and second landmarks.

A trained researcher labeled the five landmarks on US images using an open source annotation tool (LabelMe, version 4.5.9, MIT, Computer Science and Artificial Intelligence Laboratory). All labeling was confirmed by an expert radiologist or pediatric orthopedic surgeon who were familiar with the Graf method.

### Model implementation

**Fig. 2:**
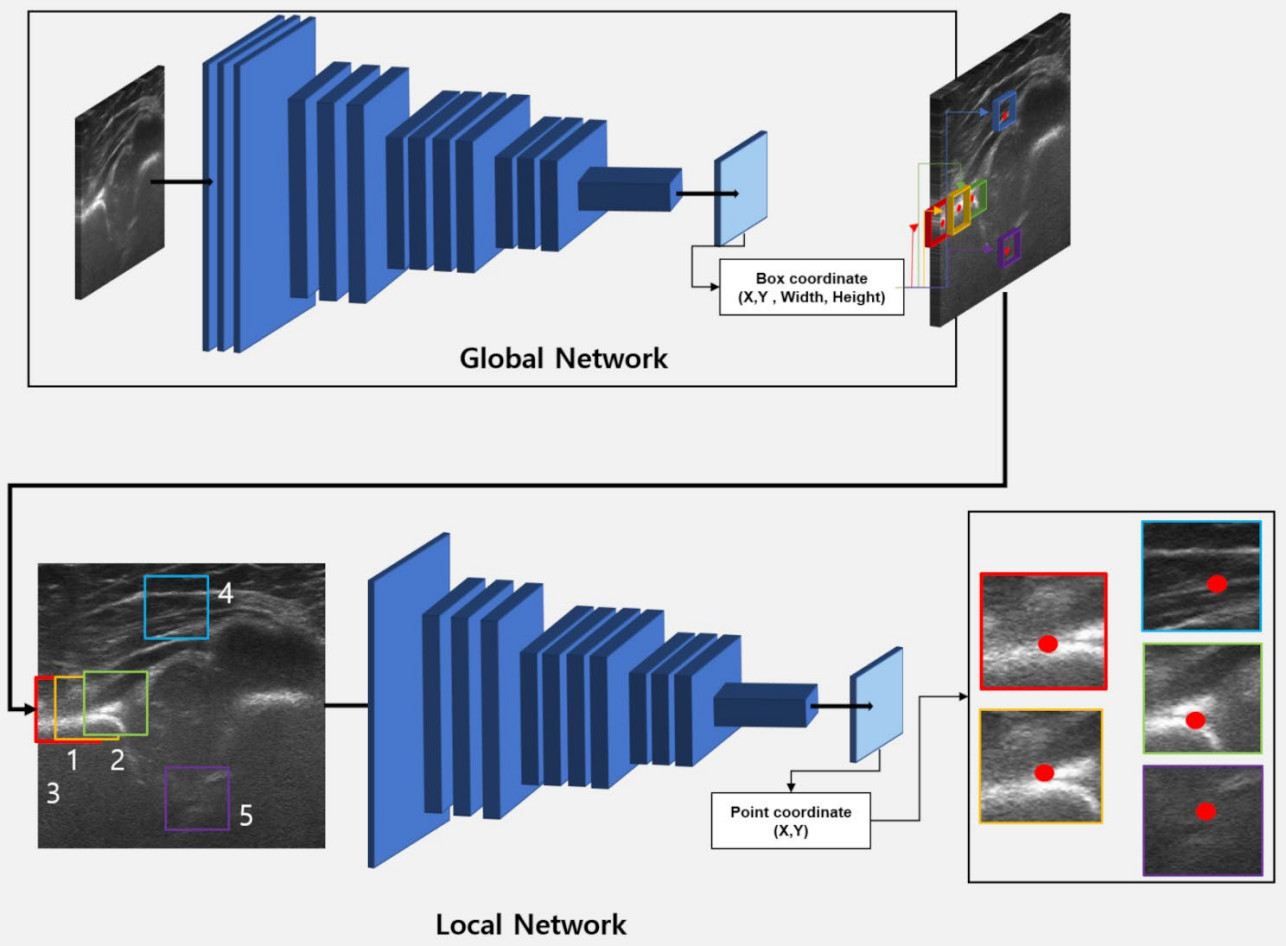
Architecture for predicting developmental dysplasia of the hip

To detect five landmarks for diagnosing and classifying DDH, local and global detection based on deep learning were performed. Our architecture-integrated global and local networks to improve landmark detection are shown in Fig. 2. All the input datasets extracted from raw dataset (US scan) were resampled to 256 × 256 pixels (XY spatial size). The gold standard is the pixel coordinate of point (red) in Fig. 2. And we generated box coordinates of 32 × 32 of the region of interest (ROI) using pixel coordinates of point (red) to train global network.

In total, 100 batches were used per epoch as shown in Fig. 2. Z-score normalization of ultrasound (US) image intensity was applied during pre-processing. We performed geometric augmentation, including zoom, rotation, and shift, on the edges of the images to improve deep-learning models against additional sources of variability. Workflows were carefully redesigned in terms of preprocessing, global and local detection based on deep leaning and computing hardware settings.

We developed integrated global and local networks based on ResNet50, EfficientNetB2, and YOLOv8. First, a global network based on deep learning architecture was developed to analyze five US landmarks [13–15]. The global network based on ResNet50, EfficientNetB2, and YOLOv8 was trained using 32 × 32 annotation in images and predicts (x, y, w, h) of box coordinates in images.

Second, a local network was developed to accurately analyze five landmarks using patch dataset (32 × 32) inferred from the global network. Additionally, we customized a last layer for dense layers to detect five landmarks, including x and y coordinates in US, and used the root mean squared error (RMSE) as a loss function (2). Lastly, the integration of global and local network is applied to detect five landmarks in US images. This model consists of a two-step structure, where the first detects ROI including specific areas in US, and the second detects five landmarks in ROI. It is to detect important landmarks of DDH using a fine-tuned model through step-by-step learning.

Various augmentations, including zoom, rotation, and shift were applied per epoch on the edges of the images to improve the robustness of the deep-learning model. These datasets were loaded on a GPU server with Ubuntu 20.04, CUDA 11.2, four 24-GB Titan RTX graphics cards, and cuDNN 10.2 (NVIDIA Corporation, Santa Clara, CA) with the PyTorch framework. We used the ADAM optimizer or the RAdam optimizer with an initial learning rate of 0.001 for landmark detection.

To train the global network, loss was calculated using the intersection of union (IOU), as presented in Eq. (1). The loss function and IOU loss (DL) are defined in Eq. (2). The volumes of the gold standard and inference for deep learning are defined by $\:{V}_{gs}\:$ and $\:{V}_{box}$, respectively.

1 $$IoU\left( {{V_{box}},\,{V_{gs}},} \right) = \frac{{2\left| {{V_{box}} \cap {V_{gs}}} \right|}}{{\left| {{V_{box}}} \right| + \left| {{V_{gs}}} \right|}}$$

The RMSE cost function in landmark detection (1.1) is provided as follows:

Additionally, the RMSE loss function (2) was used to measure the accuracy of local landmark detection for DDH using 2D US scan.

2 $$RMSE\left( {{P_g},{P_i}} \right) = \sqrt {\frac{{j = \sum\nolimits_{j = 1}^n {{{\left( {{P_{g,j}} - {P_{i,j}}} \right)}^2}} }}{n}} $$

where *P*_*g*_ is the pixel coordinate of the gold standard, and *P*_*i*_ is the pixel coordinate of the inference.

The validation errors for selecting optimized models were minimized by running the back-propagation algorithm over 25 training epochs with a batch size of eight. After training, the ablation study was conducted on the landmark detection with a test dataset to compare with other models.

The mean average precision (mAP) is used to calculate integrated result of global and local networks.

$$mAP = \frac{1}{N}\sum\nolimits_{i = 1}^n {A{P_i}} $$

where *N* is total number of classes, and $\:{AP}_{i}$ is average precision for the *i-th* class.

## Results

### Performance evaluation for detecting landmarks for DDH

First, the performance of detecting five landmarks for DDH was evaluated using 565 images through the required information of the US images (Table 1). Five landmarks 256 × 256 pixels wide, including three points in the ilium line and two points in the bony acetabulum and labrum, were predicted through global detection (Fig. 1).

The integrated results (RMSE) using the global and local networks for ResNet50, EfficientNetB2, and YOLOv8 were 4.60 ± 4.25, 4.023 ± 3.723, and 0.01 ± 0.008, respectively (Table 2). As for each point’s results, The ResNet50 results were 1.91 ± 1.30 (first point), 3.83 ± 2.44 (second point), 2.75 ± 1.63 (third point), 6.90 ± 5.30 (fourth point), and 7.63 ± 5.14 (fifth point). The EfficientNetB2 values were 1.69 ± 1.26 (first point), 3.34 ± 2.37 (second point), 2.54 ± 1.61 (third point), 5.92 ± 4.25 (fourth point), and 6.61 ± 4.82 (fifth point). Finally, the YOLOv8 values were 0.007 ± 0.006 (first point), 0.02 ± 0.002 (second point), 0.01 ± 0.008, (third point), 0.01 ± 0.003 (fourth point: ), and 0.01 ± 0.002 (fifth point).

**Fig. 3:**
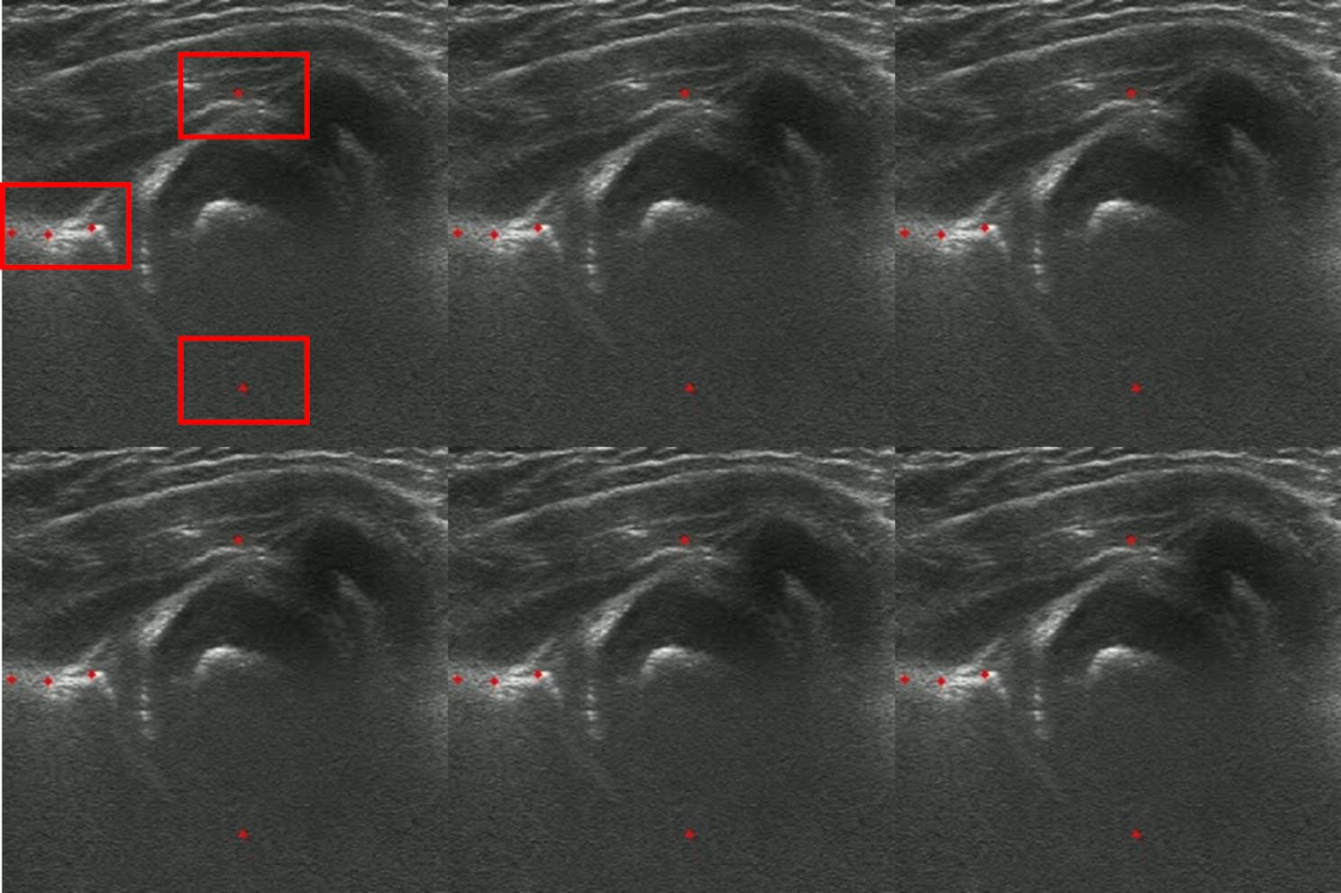
Results of developmental dysplasia of the hip prediction

Second, the integrated results [mean average precision (mAP)] using the global and local networks were 0.67, 0.74, and 0.83 for ResNet50, EfficientNetB2, and YOLOv8, respectively (Table 2). Identifying five landmarks by integrating the global and local networks is crucial for measuring the alpha and beta angles (Fig. 3).

**Table 2:** Detection of the deep-learning models for predicting the five landmarks and classifying internal and external test datasets

	Point	ResNet50	EfficientNetB2	YOLOv8
RMSE	1st	1.91 ± 1.30	1.69 ± 1.26	0.0007 ± 0.006
	2nd	3.83 ± 2.44	3.34 ± 2.37	0.02 ± 0.002
	3rd	2.75 ± 1.63	2.54 ± 1.61	0.01 ± 0.008
	4th	6.90 ± 5.30	5.92 ± 4.25	0.01 ± 0.003
	5th	7.63 ± 5.14	6.61 ± 4.82	0.01 ± 0.002
	Mean + Std	4.60 ± 4.25	4.02 ± 3.72	0.01 ± 0.008
MAP	Total points	0.67	0.74	0.83

Note: RMSE, root mean square error; MAP, mean average precision; Std, standard deviation

## Discussion

Newborn clinical screening initiatives suggest that approximately 1 out of every 100 newborns assessed exhibit signs of hip instability, although the confirmed incidence of true dislocations is documented to range between 1 and 1.5 cases per 1000 live births. Failure to diagnose DDH during the neonatal period, results in the emergence of secondary adaptive alterations, leading to compromised growth and development of the hip joint. As the age at diagnosis progresses, particularly beyond 6 months, achieving concentric reduction becomes increasingly challenging using conventional treatment modalities such as the Pavlik harness. Moreover, as the age at detection advances, the likelihood of restoring normal acetabular development diminishes. Thus, an early diagnosis with appropriate effective treatment is essential to achieve satisfactory outcomes. Considering the growing recognition of the importance of early detection before 6 months of age, ultrasonography is gaining widespread acceptance as a screening tool, particularly in assessing hips prior to femoral ossification [16].

A deep-learning neural network model system can support orthopedic surgeons in diagnosing DDH, reducing interobserver variability. In this study, we developed a model integrating global and local networks (backbone; ResNet50, EfficientNetB2, and YOLOv8) in a 2D US scan to detect five US landmarks. The best performance among of our three models was obtained using YOLOv8, both in RMSE (0.014.023 ± 0.0083.723) and mAP (0.830). In previous studies, the US images of the ilium, bony acetabulum, and labrum were segmented for automatic diagnosis, and the angles for these three landmarks were evaluated using a deep learning algorithm to obtain the alpha and beta Graf angles [10, 17–19]. Previous systems mainly focused on measuring alpha and beta angles, neglecting the assessment of plane adequacy. However, our approach diverged slightly, as we considered that obtaining an accurate US standard plane is clinically more challenging and of greater significance than the measurement of alpha and beta angles. US is a crucial tool in medicine, but the quality of the images can fluctuate significantly based on the operator’s expertise. Due to this interobserver variability, we propose that the primary focus of medical artificial intelligence (AI) applications for US should be on enhancing the examination process rather than solely concentrating on diagnosis. Our study presents a new perspective of introducing deep-learning techniques using transfer learning to detect five landmarks during US scans to achieve a reproducible standard plane. This can play a pivotal role in elevating the quality of US exams by assisting operators in image acquisition, optimization, and standardization. By doing so, AI can contribute to reducing operator-dependent discrepancies and ensure that US examinations consistently provide high-quality results and provide real-time guidance during the procedure in the clinical setting.

**Fig. 4:**
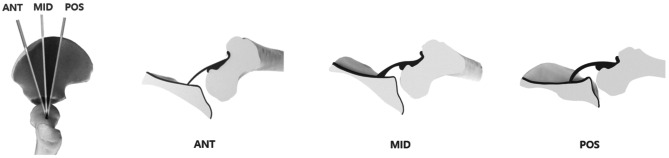
Definition of deep learning-based Graf angle measurement for predicting developmental dysplasia of the hip

According to the shape of the iliac line above the bony acetabular rim, anterior, mid (standard), and posterior sections can be distinguished from one another. In the anterior section, the silhouette of the iliac line demonstrates a gradual slope with the probe. In the midportion of the acetabular roof, the contour of the iliac line is straight and parallel to the probe. The silhouette of the iliac bone at the posterior acetabular roof demonstrates focal upward sloping (Fig. 4). However, to best of our knowledge, a guide for determining the correct imaging plane based on deep learning for hip US has not been reported. In this study, we investigated whether a deep-learning neural network model can determine the correct sectional plane on hip US using the Graf method based on pelvic landmarks. To determine the plane, the midpoint of the iliac bone was detected. Subsequently, the angle was determined as formed by two rays: one ray between the lower edge of the iliac bone and midpoint of the iliac bone, and the other ray between the midpoint of the iliac bone and acetabular bone edge. Angles between − 5° and 5° were considered standard, considering that the direction of the probe head may be different for each operator. This classification can help the operator in determining the standard plane by guiding probe movement.

However, our study had several limitations. First, we only used a single-center dataset obtained from KUAH (Table 1). A multicenter dataset is needed for externally validating the detection of the five landmarks for DDH. Second, although our model produced feasible results on identifying the five landmarks for DDH, this model should be developed further based on only deep learning using a large volume of US datasets. Moreover, comparing the final measurement between AI and experts is important to validate the performance of the model. Third, although we intended to determine the appropriate imaging plane, clinically captured US images were usually from the midline of the hip joint. Thus, anterior and posterior images of the acetabulum were relatively infrequent in our dataset. Nevertheless, our model is expected to work efficiently around the midline, which is the more important clinical location. Lastly, in order to confirm the ilium alignment, we introduced a method of measuring the angle by locating three points on the ilium bone as landmarks, but the slope of the iliac bone with the probe is not yet included in the model. In addition, the final goal should be establishing a point landmark detection algorithm and program that recognizes when the angle is parallel to the probe and captures the image in real-time in clinical settings. Further studies are necessary to compare the performance of AI-assisted and human measurements.

## Conclusions

Our deep-learning neural network model is feasible for detecting five landmarks for DDH using US images. In particular, it showed the possibility of providing clinical aid in real time in determining the standard plane for DDH screening. The measurement of primary parameters for determining DDH can be improved by applying the deep-learning model to the clinical setting. More research is needed to verify the feasibility of deep learning in infant patient positioning in other medical centers.

## Data Availability

The datasets generated and/or analyzed during the current study are not publicly available due to the nature of the image data but are available from the corresponding author on reasonable request.

## Abbreviations

AI: Artificial Intelligence
DDH: Developmental dysplasia of the hip
IOU: Intersection of Union
KUAH: Korea University Anam Hospital
RMSE: Root Mean Squared Error
US: Ultrasonography
